# Editorial

**Published:** 1864-08

**Authors:** 


### Editorial.

### SUCCESS.

The definitions applied to the term are very various. It is ordinarily defined to be the accomplishment of any special object upon which effort is concentrated. This, perhaps, is correct so far as individual objects are concerned. With those who look no farther than this it is easily attained. But success in life is a matter of far more magnitude than this. It includes the blending together in one harmonious whole, a great variety of objects and actions, and drawing from each and all the greatest and best results. Nothing less than this would we regard as success in life.

Now, what is professional success? In answering this, the rule we have just enunciated is the guide. He only can attain success, as a professional man, who lays hold upon and uses every opportunity and circumstance for the accomplishment of large and beneficent results.

Many regard it as the great object of life to accumulate money. The world says, he who has obtained wealth has made life a success; and this, too, regardless of the means by which it was obtained. This alone is *not* success.

In our profession, what is worthy of the appellation, success? Certainly not merely to gain money. Though the possession and proper employment of money is an important particular, yet it is not the chief nor one of the chief. The man who by the practice of his profession makes it his chief aim to accumulate wealth, though he does that and that only, is not successful. Neither is he successful whose highest object is to secure a large practice. One may have all this, and do comparatively little good for humanity. Neither is he successful whose aim is no higher than to become a fine operator, though it is one important element in success.

He only is successful who accomplishes, through a high and holy purpose, the largest amount of good to the greatest number of his fellow beings. Money, business, reputation and ability are all important elements of success, yet no one of them, nor all together, will constitute success in the full sense. Something beyond all these must be accomplished in order to attain perfect success.

The obligations of the dentist are three fold, viz:

Those due to himself. Those due to his patients. And those due to his profession.

Every one imagines that he can at once decide what is due to himself, and what would be best; but such decisions are aside from the mark just in proportion as the person is influenced and controlled by selfish and contracted views, opinions and practices. He whose influence for good is most outreaching and most effective is the one who secures the largest revenue of happiness. Here is the principle by which to solve the problem of our duty to ourselves.

Our duty and responsibility to our patients will be readily comprehended when we have made this first step.

However well we may conclude we fulfil our obligations to ourselves and our patients, we all must feel that our obligations to our profession are at least but poorly performed. In the fulfilment of these, we should not do it as some pay an old debt, grudgingly, and for fear lest we should over pay a few fractions; but let this work be entered upon with unbounded liberality; let every one bring in abundantly as he has been blessed, and then he shall be made fat. Let no one measure to give just an equivalent for what he has received. No one should conclude that he has but little influence, and could do but little, and therefore do nothing. If we can do but little, do *that.* Let every one use his influence and ability for the best interest of his profession—to direct it in the right channel—to make it in every sense better. No one is successful who fails in these things.

An operator, by the practice of his profession, may be the means of good to hundreds of patients, yet, in addition to this, if he can reach, and by his efforts make better operators of ten or a hundred of the professional brethren, his efforts for good to his fellow beings will be correspondingly increased.

It is the efficient employment of influence and ability in this and like channels that make the professional man successful. T.

### SUCCESSFUL TREATMENT OF AGGRAVATED DISEASE.

We not long since had the pleasure of examining some cases of disease of the mouth, in course of treatment, and some restored to health by Dr. Atkinson of New York; that far exceeded our comprehension of any bodies attainments in that direction. That alveolar abscess in its various phases, and even in very aggravated forms, is treated successfully by many in the profession we were well aware; but that such progress had been made we did not know, or conceive, till we had the demonstration.

That one, who by disease, had been rendered a loathsome, fetid, putrifying carcas, with the disease focalized in the oral cavity, could be purified and vivified, so as again to enter society, and live a life of comfort and happiness, is not according to common understanding.

Yet, we saw several cases in which this has been accomplished. Diseesed bony tissue rendered healthy and even lost portions, of considerable, extent, reproduced, giving the original form and simetery to the parts; and soft parts restored to a healthy condition, even when verging on the point of dissolution, and reproduced when lost. Now although this is almost beyond belief, yet it is accomplished by such means as are available to all, who have the inclination and ability to make them theirs.

These attainments are gained by a long thorough and deep study and investigation into the laws of life, health and disease, and an understanding of those agents, instrumentalities and circumstances, that influence and modify these conditions. Attainments of this kind are the results of ardious labor and investigations of almost a lifetime, such as most persons do not care to enter upon.

To Dr. A. certainly belongs the high regard, and real gratitude of our profession, for that which he has accomplished, for most assuredly it has not came spontaneously. T.

### PERSONAL.

We had a letter from our assistant editor, Dr. Watt, a day or two ago. He reports that his health has very much improved. Has not had sick headache (with which he was very much afflicted before) since he has been in the service; has "consolidated from 211 lbs. to 180! laying off his pomposity." Says he has been shot at several times but not hit. May they never hit him. His health will doubtless be greatly improved—by going to war—which we know will be a matter of gratification and joy to the readers of the *Register*. T.

### WABASH VALLEY DENTAL ASSOCIATION.

Order of Business and subjects for discussion at the October Meeting of the Wabash Valley Dental Association to be held at Crawfordsville, October 25, 1864.

### Order of Business.

1st. Report of Committees.
2d. Admission of Members.
3d, Reading of President's Address.
4th, Reading of Papers.

### Discussion of the following Subjects.

1st. Filling Teeth, Simple and Complicated Cavities.
2d. Treatment of Dental Pulp.
3d. Alveolar Abscess, its Treatment.

### Mechanical Dentistry.

1st. Artificial Dentures, temporary and permanent.

### Miscellaneous Subjects.

1st. Anæsthetics.
2d Professional Etiquette.
3d. Unfinished Business.

J. W. FAHNESTOCK,

Committee on order of Business.

